# The Effect of Theory-Based HIV/AIDS Educational Program on Preventive Behaviors Among Female Adolescents in Tehran: A Randomized Controlled Trial

**Published:** 2020

**Authors:** Farideh Khalajabadi Farahani, Fatemeh Darabi, Mehdi Yaseri

**Affiliations:** 1- Department of Population and Health, National Population Studies and Comprehensive Management Institute, Tehran, Iran; 2- Department of Public Health, Asadabad School of Medical Sciences, Asadabad, Iran; 3- Department of Epidemiology and Biostatistics, Tehran University of Medical Sciences, Tehran, Iran

**Keywords:** Adolescents, Education, HIV/AIDS, Theory of planned behavior

## Abstract

**Background:**

Adolescents are increasingly at risks of HIV infection through high-risk sexual behaviors. This paper aimed to assess the effectiveness of a HIV/AIDS educational intervention among female adolescents in Tehran.

**Methods:**

A randomized controlled trial was conducted among high school girls aged 12–16 years studying in Tehran in 2016–17. The sample was selected using multistage random cluster sampling. Four schools per district were randomly selected using probability-proportional to size. Adolescents were randomly assigned to the experimental (n=289) and the control (n=289) groups. The theory of planned behavior (TPB) was the basis of both the intervention and the evaluation phase. Both experimental and control groups completed a questionnaire designed for HIV related behaviors for female adolescents (HBQFA) at baseline and after six months follow up. A theory-based educational program was implemented for the experimental group.

**Results:**

The mean age of participants was 14.1 years (SD=0.96). In the experimental group, significant improvements were shown in adolescents’ HIV knowledge (31.9%, 95% CI: 28.8–35.0), attitudes towards HIV (16.6%, 95% CI=14.4–18.8), subjective norm (16.8%, 95% CI=12.9–20.6) and perceived behavioral control (19.1%, 95% CI=16.2–22.1), perceived parental support (17%, 95% CI=13.8–20.2), behavioral intention to prevent HIV (19%, 95% CI=16.3–21.6), and HIV preventive behavior (17.3%, 95% CI=13.9–20.6) (p<0.001).

**Conclusion:**

Theory-based educational intervention on HIV/AIDS prevention can significantly protect adolescents from misconceptions, wrong attitudes and risky behaviors and unsupportive social environment that expose them to greater HIV risk. Health policy -makers are advised to consider effective training programs related to HIV/AIDS prevention behaviors in the school system.

## Introduction

According to 2016 Iranian population and housing census, there were approximately 11 million adolescents aged 10–19 across the country (1). Adolescents and young people are at greater risk of HIV and account for about half of the new HIV infections (2). Adolescents are in an important period of physical, mental and social development and they are in a critical stage for development of healthy beliefs and practices including HIV preventive behaviors. Scientific evidence shows that many young people are at risk of HIV infection because of their risky sexual behavior (3, 4).

Nearly 25% of new HIV infections occur among people younger than 22 years (5). Young people are typically identified to be at the starting line of the AIDS pandemic regardless of whether they live in concentrated or generalized epidemic countries (6). Particularly, adolescents in conservative societies who are not targeted for any education on sexual health, tend to be unknowledgeable about HIV risks and tend to be more vulnerable to high risk sexual behaviors. This makes HIV educational interventions imperative particularly among young generation (7).

Evidence also shows that adolescents in digital era have increasingly access to sexually explicit materials in internet including pornography and are more likely involved in sexual relationships before marriage (8). A significant minorities of young men in metropolitan areas of Iran engage in premarital sex (4). The corresponding rate is much lower among females due to gender based double-standards or partly due to under reporting. Such involvement in premarital sex might be due to different factors such as socio-economic changes including delay of marriage (9) and access to global media and communication technology and changes in sexual values. While multiple partners and lack of consistent condom use are prevalent among youth that are sexually active (10, 11), important misperceptions about HIV/AIDS prevail (12). This exposes youth at greater sexual health risks (13). In fact, many social and cultural barriers prevent introducing HIV and sexual health education for unmarried people in conservative societies such as Iran (14, 15).

At the global level, five youth per minute and 6000 people per day get infected with HIV (16). In Iran also, 46.5% of the HIV positive people were in the 25–34 year-old age group (17). Despite the fact that HIV rate in general population of Iran is lower than 0.1% and the infection is concentrated mainly among high risk groups such as injection drug use (2), the mode of HIV transmission is shifting from unsafe injection to unsafe sex in recent years. From among HIV positive identified between 1986 to 2016, in about 66% of cases, the mode of transmission was injection drug use and only 19.6% was unsafe sex, while during March, April and May 2017, the corresponding rates changed into 33% and 48%, respectively. That means HIV incidence due to unsafe sex has doubled from 19.6% to 47.9% which is a concerning trend and needs serious educational intervention for young population (18).

Since 20% of registered HIV positive people are between the ages of 20–29 years (18) and because of long incubation period for HIV, it is likely that infections happen during the teen years and young ages. Pornography access has also provided a platform for sexual excitement among youth (19). Young women in cultures with strong emphasis on physical virginity become more vulnerable towards HIV and STIs if they get involved in premarital sex. They tend to practice unprotected anal and oral sex to preserve their virginity while are not concerned about HIV. Condom is mainly used by young people to prevent pregnancy not STIs and HIV (20). Moreover, the rate of HIV infection among women in 2017 has doubled compared to the rate between 1986 to 2016 (21).

**Figure 1.**
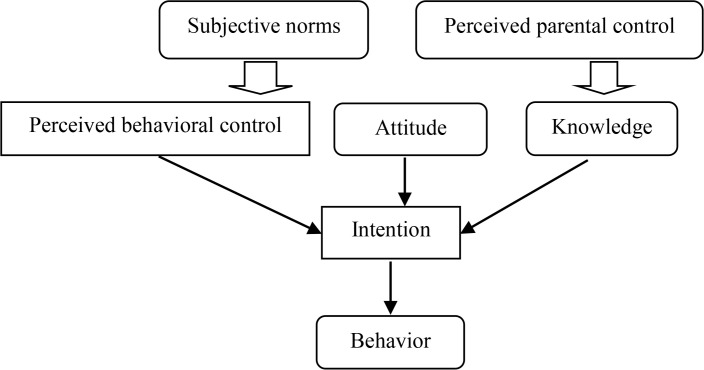
Conceptual framework based on modified theory of planned behavior

A considerable body of research has confirmed the power of the TPB to predict intentions and behaviors across a range of health behaviors (22). Prior to assessing HIV preventive behaviors, assessing one’s knowledge, attitudes, and self-efficacy are necessary (4). Since majority of health problems including HIV/AIDS are bonded with human behaviors, it is necessary to employ behavioral theories in designing HIV intervention (23). According to the theory of planned behavior (TBP), attitude, subjective norms, and self-efficacy are important determinants of motivation for carrying out a certain behavior which protects the individual from HIV risks (24). The individual’s attitude towards learning about HIV/AIDS predominantly derives from a set of beliefs about the negative and positive outcomes of HIV prevention. Subjective norms refer to approval or disapproval of significant others (Like fellow students or teachers) towards HIV/AIDS prevention. Finally, perceived behavioral control refers to the confidence of being capable of performing the behaviors required for HIV/AIDS prevention, like open communication about HIV/AIDS in a class. In this study, the students’ perception of parental control (4) was also added to the conceptual model (Figure 1).

This study aimed to assess the effectiveness of a theory-based HIV/AIDS educational intervention in HIV prevention among female adolescents in Tehran. Specific objectives include assessing the effect of such school based educational intervention on HIV knowledge, attitude, social norms, behavioral control, parents support, intention and behavior among female adolescents.

## Methods

### Study design:

A randomized controlled trial (RCT) was conducted among female students aged 12–16 years of selected secondary public schools in Tehran, the capital of Iran, from October 2016 to May 2017. Initially, a pretest was performed for both experimental and control groups, then educational program was implemented for intervention group, and after 6 months of follow up, changes in various constructs of TPB were assessed in both control and experimental groups. Public schools are governmental and free of charge in Iran. Private or non-governmental schools were not considered in this study.

The sample size was calculated based on HIV knowledge rate derived from previous studies with a power of 90% and a 10% attrition rate considering the design effect of two and having 10% loss to follow up. Sample size was calculated based on the difference between the knowledge score that produced the maximum sample (25, 26). For this purpose, the mean sample size in the control group was 59 (22.9%) and the intervention group 85.9 (25.2%) obtained from similar studies and from the score of 100.

Eventually, 289 students were enrolled in each group of experimental and control (n=578).

This RCT was approved by the ethic committee of Tehran University of Medical Sciences (Ethics code No. 651, dated Monday, April 25, 2016) and registered as clinical trials in Iran on November 13, 2015, (IRCT2015070623089N2 code). The principal investigator provided adequate information about the project and its aims to the students. They were asked to bring their parents’ informed consent to participate in this study emphasizing that their participation was completely voluntary. They were assured of the confidentiality and anonymity of their responses.

### Setting and participants:

To collect a representative sample of students from among public high schools in Tehran, and avoiding selection bias, three districts out of 22 municipal districts were selected using proportional stratified random sampling. Four schools per district were randomly selected using probability-proportional to size. Then, three classes per school and 16 students per class were randomly selected. The block stratified cluster randomization method was used, and samples were selected separately in every district. From among the four selected schools per district, two were assigned to the experimental group and the other two were assigned to the control group. A detailed description of participant’s enrollment, group allocation, and follow-up can be found in a previous publication of this research (4).

**Figure 2.**
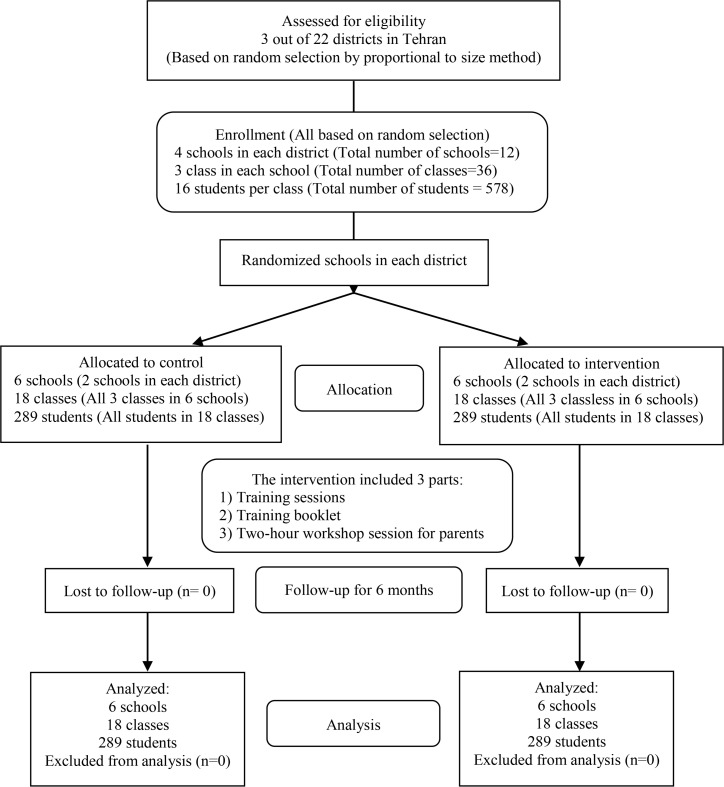
Flowchart of sampling procedure for the interventional study

In order to match the participants in the experimental and control group before the intervention, some important socio-economic characteristics were compared to make sure that they were not significantly different. Some of these characteristics were “age, family size, economic status, father and mother’s education”. There was no significant difference in socio-economic situation of experimental and control group. The mean score of attitude, perceived norms, behavioral intention, behavioral control, parental control, knowledge and behavior were also not statistically different between the experimental and the control group (Figure 2).

### Intervention module:

The educational intervention was implemented based on the theoretical framework. It was comprised of education about HIV virus, its risk factors, HIV prevention, modes of HIV transmission, and risky behaviors such as HIV transmission by infected blood, tattoos, unsafe injection among drug users, high risk sexual practices such as unprotected sex and anal intercourse. The training was implemented in six different phases (Each phase took 2 *hr* with a 20-*min* break). In each phase, one special educational goal was followed. The educational goals for each phase were as follows: Phase 1; rise in awareness towards HIV/AIDS infection, Phase 2; creating a positive attitude towards learning about HIV/AIDS, Phase 3; approval or disapproval of significant others (Like fellow students or teachers) towards learning about HIV/AIDS, Phase 4; influence of parents as reinforcing agents to support students on learning about HIV/AIDS prevention, Phase 5; self control of self-efficacy refers to the confidence of being capable of performing the behaviors required for learning about HIV/AIDS, and finally, Phase 6; skill needed for HIV/AIDS prevention.

Some educational strategies were employed to achieve the behavioral goals in each phase. The aim was also to correct some misperceptions about transmission of HIV/AIDS through mosquito bites, shared swimming pools and bathroom, and also reduce stigma and create supporting attitudes towards people who live with HIV. Training program is detailed in table 1.

**Table 1.** The format and content of the HIV/AIDS educational program

**Training phase (each educational goal was held in 4 sessions)**	**Goal**	**Type of training**	**Number of sessions and duration**
**Phase 1**	Increasing awareness of HIV/AIDS	- Lectures using PowerPoint slides - Small group discussion	4 sessions, each 2 *hr* (8 *hr* total )
**Phase 2**	Creating positive attitude towards learning about HIV/AIDS	- Small group discussion - Brainstorming - Story-writing - Question and answer sessions with feedback from the students	4 sessions, each 2 *hr* (8 *hr* total)
**Phase 3**	Approval or disapproval of significant others (Like fellow students or teachers) towards learning about HIV/AIDS	- Small group discussion - HIV/AIDS prevention skills education - Decision making and brainstorming	4 sessions, each 2 *hr* (8 *hr* total)
**Phase 4 (Intervention for parents)**	Influence of parents as reinforcing agents to support students on learning about HIV/AIDS prevention	- Workshop for parents official invitation of parents to attend the school and participate in a workshop which took 4 *hr*	4 sessions, each 2 *hr* (8 *hr* total)
**Phase 5**	Self-efficacy refers to the confidence of being capable of performing the behaviors required for learning about HIV/AIDS	- Discussion forums - Interactive discussion	4 sessions, each 2 *hr* (8 *hr* total)
**Phase 6**	Skill regarding HIV/AIDS prevention	- Lectures, questions and answers, small group discussion	2 sessions, each 2 *hr* (4 *hr* total)

The intervention was performed by the principal investigator (The 2nd author), a female professional in health promotion. A total of 289 female participants in the intervention group received the module which lasted about three months. A booklet was developed for adolescents aged 12–16 years. In addition, the final draft of the booklet was evaluated by adolescents outside the schools before applying to the current study. The booklet entitled “Training HIV/AIDS Prevention” addressing adolescents and shedding light on their importance. The introductory section outlined the content of the booklet. The other sections were based on the TPB model constructs using simple language.

Six months after the intervention, students in both groups were followed up and using a questionnaire, their changes in different constructs of TPB were assessed. For ethical consideration, the control group also received the educational materials when the study was finalized. Interestingly, since the principal investigator was in charge of the training and data collection and due to her emphasis on the presence of students in all training sessions and follow up data collection, there was no missing data after the intervention and in the follow up phases.

### Measures:

A valid, pretested and self- administered questionnaire consisting of seven sections was employed to evaluate the effectiveness of the intervention. Data were collected at the baseline, and six months after the intervention from both groups. The data was collected from September 2015 to July 2016. The inclusion criterion was comprised of “willingness to participate, full participation in training sessions and attending post intervention assessment”. No one was excluded from this study. The instrument was comprised of 34 questions and items were derived from the World Health Organization questionnaire (27), the literature review, and a qualitative study conducted before the intervention. Another part of the instrument was developed by literature review and a qualitative study together with eight focus group discussions (FGDs) with 20 participants. Different constructs of TPB including attitude, subjective norm, behavioral control, behavioral intention, perceived parental control and HIV/AIDS knowledge and preventive behavior were included in the study tool.

### Variables and constructs:

The questionnaire was structured in three sections:

1. The demographic information including age, mother’s job, father’s job, and father’s education, mother’s education and financial situation (Good, average and weak).
2. General knowledge about HIV/AIDS: sixteen items assessed the participants’ knowledge about HIV/AIDS. For instance, “HIV is transmitted through unprotected sex”. The responses comprised “true”, “no”, “do not know”. Each correct answer was given one score, and wrong and “do not know” responses were scored zero. Based on these items, a scale of HIV knowledge was developed.
3. Modified TPB section: Attitudes towards HIV were measured by five statements in a 5-point Likert scale ranging from strongly agree (Score 5) to strongly disagree (Score 1). For example, “in my opinion, HIV/AIDS is a serious problem for the health of all people”. Perceived behavioral control was measured by 3 items in a 5-point Likert scale ranging from completely confident (Score 5) to not at all confident (Score 1). For example, “I’m sure that I can avoid risky behaviors such as unprotected sex and tattoos, which transmit HIV/AIDS”. Behavioral intention towards HIV/AIDS was a scale which comprises 3 items in a 5-point Likert scale ranging from always (Score 5) to never (Score 1). For instance, “I’m going to talk about problems such as sexuality and HIV/AIDS with my parents and family members”. This scale indicates the respondents’ intention to communicate with peers and family about HIV/AIDS and intention to learn about HIV. Finally, two items were used to assess HIV/AIDS preventive behaviors, *e.g*., “I avoid having contact with sharp objects such as HIV/AIDS contaminated syringes, and needles infected with HIV/AIDS”. Overall knowledge was determined by aggregating correct answers from all knowledge questions (0–100 for questions on HIV/AIDS knowledge). Due to different number of items for each scale, all scales were transformed into the scale of 0 to 100 to be comparable. Three levels of low (0, 33), medium (34–66), and high (67–100) were specified. Validity and reliability of the study tool was assessed and its details have been previously published (28).

In this study, to assess the validity of the scales, face validity, content validity, and construct validity were assessed. In the qualitative face validity, participants stated that they had no problems with reading and understanding the items. The numeric value of content validity ratio (CVR) was 0.64, determined by the Lawshe table. An ordinal scale with four possible responses was used to obtain content validity index (CVI) for relevance, simplicity, and clarity of each item. The mean CVI was 0.74. The responses ranged from 1=not relevant to 4=very relevant. The number of those judging the items as relevant or clear was determined by the content experts. The constructed questionnaire was evaluated for validity by exploratory and confirmatory factor analysis (EFA and CFA) and to assess the reliability of the scale, the internal consistency and stability of the scale were measured (Table 2).

**Table 2.** Number of items, range, mean and standard deviation and alpha Cronbach of the TPB constructs (n=578)

**TBP scales**	**No. items**	**Range**	**Mean±SD**	**Alpha cronbach**
**Subjective norm**	3	0 to 100	33.0±20.2	0.89
**Perceived parental support**	2	0 to 87.5	45.1±15.1	0.94
**HIV/AIDS knowledge**	16	0 – 100	47.1±20.1	0.87
**Attitudes towards HIV/AIDS**	5	0 to 80	48.0±9.4	0.85
**Perceived behavioral control**	3	0 to 92	34.2±15.1	0.81
**Behavioral intention**	3	0 to 100	33.4±12.8	0.80
**Preventive behavior**	2	0 to 100	42.9±16.1	0.89
**Total**	34	0 to 100	40.6±7.9	0.94

### Statistical analysis:

To make the results comparable, the scores of all constructs were converted to scores from 0 to 100. A series of analysis of variances and Chi-square tests on socio-demographic characteristics and baseline measures were conducted to make sure that the random allocation has been successful. The differences between pre-tests and post-tests in the intervention group and those of the control group have been compared using paired t-tests. The comparison between scores of scales in the intervention group with that of the control group was implemented using paired t-tests because the outcome variables were numerical measures. Linear regression models were used (Ordinary least squares) to estimate intent-to-treat effects on different constructs of TBP. The SPSS software version 23 was used for analysis. A p-value of 0.05 was chosen to reject the null hypothesis for each comparison.

To consider the correlation of measurements in the clusters, multilevel analysis was used. The likelihood ratio (LR) was used to acquire the proper number of levels in multilevel analysis. The comparison of the baseline values between the two groups was performed by a two-level multilevel analysis. The three-level multilevel analysis was used to evaluate the changes in each group. To compare the changes in the scores of the dimensions between the two groups, the interaction between the groups and time in a multilevel analysis was examined. To assess the deterministic role of the educational intervention in behavior improvement, the role of other significant factors like knowledge was controlled and some other multilevel analyses using different sets of explanatory variables were used. At long last, multivariate analysis of variance was used simultaneously to evaluate the effect of education on improving all dimensions. All statistical tests were two-sided, and p-values were considered statistically significant if lower than 0.05. Data were analyzed using SPSS software, version 23.

## Results

There was no significant difference at baseline characteristics of the experimental and control group. In total, the mean age of participants was 14.1. The number of educational years for their fathers and mothers was between 6–12 years by the majority (About 64% and 66%). In total, the majority of fathers were employed (94%), while over two thirds of mothers were housewives (73.5%). About 85% of students reported their financial situations as “good and average” (Table 3).

**Table 3.** Socioeconomic characteristics of the participants (n=578)

		**Total n (%)**	**Group**		**p-value**

			**Experimental n (%)**	**Control n (%)**	
**Age (mean, SD)**		14.1 (0.96)	14.02 (0.99)	14.2 (0.91)	0.473
**Father’s education (years)**					
	<6	57 (9.9%)	34 (11.8%)	23 (8.1%)	0.291
	6–12	366 (63.8%)	191 (66.1%)	175 (61.4%)	
	>12	151 (26.3%)	64 (22.1%)	87 (30.5%)	
**Mother’s education (years)**					
	<6	72 (12.5%)	35 (12.1%)	37 (12.9%)	0.427
	6–12	379 (65.8%)	204 (70.6%)	175 (61.0%)	
	>12	125 (21.7%)	50 (17.3%)	75 (26.1%)	
**Father’s employment status**					
	Employed	544 (94.1%)	275 (95.2%)	269 (93.1%)	0.297
	Unemployed	34 (5.9%)	14 (4.8%)	20 (6.9%)	
**Mother’s employment status**					
	Employed	153 (26.5%)	74 (25.6%)	79 (27.3%)	0.637
	Housewife	425 (73.5%)	215 (74.4%)	210 (72.7%)	
**Financial condition (self-reported)**					
	Very good	35 (6.1%)	12 (4.2%)	23 (8.0%)	0.442
	Good	242 (41.9%)	114 (39.4%)	128 (44.3%)	
	Average	254 (43.9%)	131 (45.3%)	123 (42.6%)	
	Weak	47 (8.1%)	32 (11.1%)	15 (5.2%)	

The finding showed that students’ awareness of HIV/AIDS before the intervention was moderately poor about HIV transmission through breast milk, infected syringes and needles, blood, injection drug use and unprotected sex. Before the intervention, about 32% of female students correctly knew that HIV can be transmitted through blood. Only 23% knew that it can also be transmitted through infected instruments such as syringes and needles, about 37% knew that HIV can be transmitted through needles in injection drug users, about 48% knew that HIV can be transmitted through shaving/tattoo, while only 36% knew that it can be transmitted through unprotected sex with infected partner. Only about 20% knew that HIV can be transmitted through breast milk. Importantly, there was a huge misperception about HIV transmition through sneeze and cough (57%) or through kissing (62.3%). About 34% had a misperception that HIV can be transmitted through swimming pools and bathroom. Moreover, 47% had a misperception that HIV can be transmitted by mosquitoes’ bites. As low as 20% knew that HIV test is the only mean to diagnose AIDS.

The scores of different constructs of TBP before and after the intervention for both experimental and control groups are presented in table 4. In post intervention phase compared to the baseline, a significant improvement was observed in the scores of knowledge and all other TPB-constructs (p<0.001). Despite lack of significant difference in the mean scores of knowledge between experimental and control at baseline (34.3±26.8 and 34.3±26.8, p>0.99, respectively), a significant improvement occurred after the intervention in the experimental group compared to the control group (86.3±14.4, *vs*. 52.3±22.5, p<0.001). In addition, a significant improvement occurred in the attitude score after the educational intervention in the experimental group compared to the control group (89.4±7.5, *vs*. 70.8±11.9, p<0.001).

**Table 4.** The mean score of knowledge and TPB constructs on HIV/AIDS among adolescent females at pre-intervention and post-intervention

		**Group**		**Diff**	**95% CI**		**p**
		**Intervention**	**Control**		**Lower**	**Upper**	
**HIV knowledge**							
	Pre	47.1±20.1	47.1±20.2	0.0	−3.3	3.3	>0.99 ^‡^
	Post	83.9±14.1	52.0±22.6	31.9	28.8	35.0	<0.001 ^§^
	change	36.8±24.0	4.92±23.7	31.9	28.8	35.0	
	P-within ^‡^	<0.001	<0.001				
**Attitude**							
	Pre	49±9.3	47±9.4	1.9	0.4	3.4	0.014 ^‡^
	Post	89.4±7.5	70.8±11.9	14.7	13.1	16.3	<0.001 ^§^
	change	40.4±12.2	23.8±14.9	16.6	14.4	18.8	
	P-within ^‡^	<0.001	<0.001				
**Subjective norm**							
	Pre	33.1±20.2	33.1±20.2	0.0	−3.3	3.3	>0.99 ^‡^
	Post	60.8±8.7	55.2±13.4	16.8	14.9	18.6	<0.001 ^§^
	change	27.7±21.7	10.9±25.4	16.8	12.9	20.6	
	P-within ^‡^	<0.001	<0.001				
**Behavioral intention**							
	Pre	33.8±12.6	33±13	0.9	−1.2	3.0	0.416 ^‡^
	Post	62.1±7.1	42.3±11.9	18.1	16.5	19.7	<0.001 ^§^
	change	28.3±14.6	9.3±17.9	19.0	16.3	21.6	
	P-within ^‡^	<0.001	<0.001				
**Behavioral control**							
	Pre	33.9±14.9	34.5±15.3	−0.6	−3.1	1.8	0.614 ^‡^
	Post	64.8±6.5	46.2±12.3	19.8	18.2	21.4	<0.001 ^§^
	change	30.9±16.2	11.7±20.2	19.1	16.2	22.1	
	P-within ^‡^	<0.001	<0.001				
**Behavioral parent support**							
	Pre	44.2±14.9	45.9±15.3	−1.7	−4.2	0.8	0.18 ^‡^
	Post	88.2±11.1	66.9±14.3	18.7	16.6	20.8	<0.001 ^§^
	change	38±18.2	21±20.8	17.0	13.8	20.2	
	P-within ^‡^	<0.001	<0.001				
**Preventive behavior**							
	Pre	41.8±16.1	44.2±16	−2.4	−5.0	0.2	0.075 ^‡^
	Post	76.6±11.3	61.7±13	19.6	17.6	21.6	<0.001 ^§^
	change	34.8±20	17.5±20.7	17.3	13.9	20.6	
	P-within ^‡^	<0.001	<0.001				

$ p-value for interaction between the groups and time, based on a multilevel analysis

‡ p-value for change based on the linear mixed model

While there was no significant difference in the mean score of subjective norm between the intervention and the control group at baseline (Mean= 33.1±20.2, control=33.1±20.2, p=0.99, respectively), a significant improvement occurred in the mean score of subjective norms after the educational intervention in the experimental group compared to the control group (60.8±8.7, *vs*. 55.2± 13.4, p<0.001). Similarly, a significant improvement occurred in the score of perceived behavioral control after the educational intervention in the experimental group compared to the control group (64.8±6.5, *vs*. 46.2±12.3, p<0.001).

**Figure 3.**
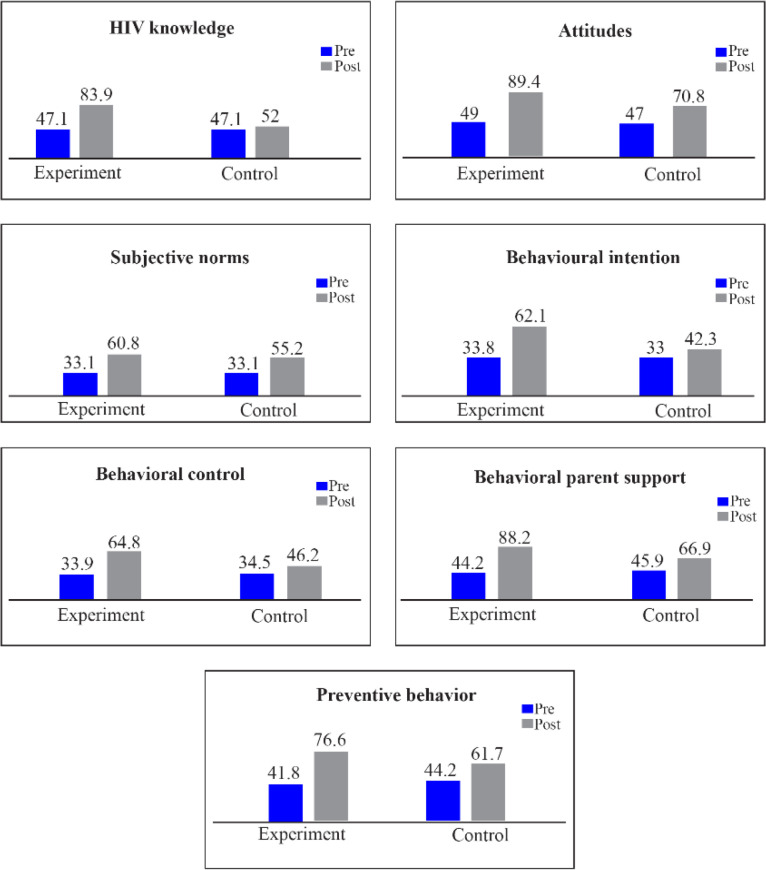
Mean scores of different TPB constructs pre and post intervention in experimental and control group

Regarding perceived behavioral control, also a significant improvement was seen in the score after the intervention in the experimental group compared to the control group (88.2±11.1, *vs*. 66.9±14.3, p<0.001). The score of intention was improved significantly after the intervention in the experimental group compared to the control group (Mean score: 62.1±7.1, *vs*. 42.3±11.9, p<0.001). Finally, improvement in preventive behavior was shown after the educational intervention in the experimental group compared to the control group (76.6±11.3, *vs*. 61.7±13.0, p<0.001) and the size of improvement in the experiment group was considerably greater than the corresponidng rate in the control group (34.8±20.0 *vs*. 17.5±20.7). It should be noted that although the score of most constructs of TPB showed an increasing change from pre-intervention to post-intervention in both groups, the size of change was significantly greater in the experiment group compared to the control group (Table 4, Figure 3).

## Discussion

This study employed an extended version of the TPB framework to evaluate the effectiveness of an educational program on HIV prevention among female adolescents using a randomized control trial in Tehran. Female students were poorly informed of HIV/AIDS before the intervention, and many misperceptions about HIV prevailed among adolescents which was consistent with other previous studies (4, 29, 30). Misinformation on HIV among adolescents is due to the lack of formal and comprehensive education on HIV/AIDS in Iran. In fact, It is not a major issue in students’ daily lives, and it can be due to the complexity of the topic and receiving contradictory information on the prevention, treatments and transmission mode by their parents, schools and media (31). This study revealed that a school based educational intervention based on a behavior change theory such as the theory of planned behavior can be effective in HIV prevention among adolescents and young people.

Significant changes in all different constructs of TPB were indicated showing that a school-based HIV educational program can be practical and effective in changing HIV behaviors among adolescents. In fact, the observed changes in the outcome variables are attributed to the impact of the educational intervention. These results will have important implications in designing and implementing HIV prevention programs for adolescents especially in schools. Such results are consistent with previous evidences regarding the effect of TPB-based educational intervention on improvement of HIV knowledge and behaviors (4, 32, 33).

The TPB suggests that changing intentions can be accomplished by influencing attitudes, subjective norms and perceived behavioral control. Thus, to design effective interventions, the relative importance of beliefs, social norms and perceived behavioral control in explaining behavioral intention becomes vital (34).

The intervention in this study led to a significant improvemnet in attitudes towards HIV, as well. Before the educational intervention, most adolesents believed that HIV/AIDS is a serious problem for other people and not a concern in school. However, after the intervention, majority of students changed their mind and believed that HIV is a serious problem for the wellbeing of all people. Beliefs are the core of understanding one’s intention to do or abstain from AIDS-related behavior. Most HIV prevention programs target one’s attitude towards HIV. This study showed that educational intervention affects students’ attitude and enables them to establish more toleration toward HIV-positive people as well. The fact that HIV positive people should not be isolated from the school and workplaces was developed by such interventions. These results are consistent with the findings of other studies (33, 35).

Based on this study, the intervention had also a significant effect on perceived social norms among students regarding HIV prevention which is consistent with previous evidence (4). Due to the fact that the intervention was conducted in the school setting, it is very likely that such changes are due to indirect influence of such education on other students and moderation of norms. In this study, HIV/AIDS was considered primarily a social problem by students. Approximately, half of the students showed an avoidance of sitting near an infected student. Hence, it seems that involvement of students with the subject and communication with parents, researcher and friends about HIV would have some influences upon subjective norms on HIV among adolescents. Perceived social norms is an important factor for avoiding risky behaviors such as unprotected sex, injection of contaminated blood, and tattoos. Hence, perceived social norms need to be considered while designing a school-based intervention. Attempt at improving social norms toward HIV, peer education, friends, parents and teachers have a positive impact on HIV/AIDS prevention and high risk behaviors among adolescents.

In addition, our intervention had an effect on the improvement of the perceived behavioral control among students toward HIV/AIDS. This finding was also consistent with other researches by Albarracin et al. (36) and Kaveh et al. (30). In conservative societies such as Iran, where open communication about sexual matters in the national media and in the society is prohibited, no formal and explicit education on sexual health and HIV/AIDS exists for adolescents and low behavioral control among adolescents will expose them at greater risks of HIV/AIDS if they get involved in high risk behaviors. Self-efficacy helps individuals to be able to reject high risk behaviors such as unprotected sex, and unsafe injections, etc. The relationship between higher self-efficacy and HIV preventive behavior has been confirmed before (4).

The findings of this study suggest that parents can play a critical role in HIV/AIDS preventive behaviors. Parents support is critical for adolescents to seek proper information about HIV/AIDS and also have safe behaviors. Therefore, HIV intervention requires defining the specific role of family and parents in order to be able to change risk behaviors. School-based HIV education which involves parents as an acceptable source of support has been considered a social vaccine (37). Parents can influence the health of their children by establishing close and intimate relationships with their children and educating them about high-risk behaviors, including unsafe sex, unsafe injection, tattoos, and so on. Consistent with other studies (4, 30), the current study indicated the significant improvement of parental control in the experimental group compared to the control group.

Finally, this study demonstrated a significant improvement in behavioral intention for prevention of HIV and also preventive behaviors. However, more longitudinal studies are needed to evaluate behavioral changes during a longer period.

### Limitations and strengths:

This study suffered from some limitations. First, due to the conservative nature of the society and the school environment, the researchers were restricted in asking sensitive questions with sexually explicit content in school system such as “anal sex” or “oral sex” or “condom use”. Many misperceptions might be related to these types of non vaginal sex and about protective role of condom in different types of sex. In addition, these results can only be generalized to female adolescents who study in public schools in urban areas in Tehran, the capital of Iran, and similar students in similar societies. Therefore, one should be cautious with the generalization of results. Male students and students from private sector or nonprofit schools might be different and they need to be researched separately. It is also unclear that these changes in constructs of the TPB theory are sustainable or not. Perhaps, interventions need to be repeated with some intervals which need to be studied in longitudinal researches.

Moreover, most constructs of TPB were improved in the control group as well; one possible explanation could be the curiosity of the teenagers after the exposure to the questions about HIV and exchange of knowledge among students in intervention group with other peers or search of information on HIV. However, the size of change in the intervention group was much greater than that of the control group. Longitudinal studies are suggested for future to be able to assess students’ high risk and protective behaviors related to HIV risk between experimental and control group. This study benefited from some strength including face-to-face education and having a control group and equal chance of being selected in the experimental or control groups in a randomized control trial (RCT).

## Conclusion

The study shows that HIV/AIDS preventive educational programs based on the theory of planned behavior are effective and beneficial in improving HIV prevention knowledge, attitude, subjective norms, perceived behavioral control and preventive behavior among in-school adolescents. HIV/AIDS educational programs can protect adolescents from wrong attitudes and risky behaviors and unsupportive social environment that expose them to HIV/AIDS risk, especially where adolescents are at risk of HIV infection. HIV/AIDS education will be more successful if it employs long-term strategies for adolescents. School-based HIV/AIDS education is a critical tool for prevention of high risk behaviors among adolescents. Finally, similar interventions should be conducted on both genders and other students in other provinces.
